# Supplementary material for: Genome-Wide Identification and Expression Analysis of the ClHMGB Gene Family in Watermelon Under Abiotic Stress and Fusarium oxysporum Infection
Source: Int J Mol Sci. 2025 Dec 23;27(1):157. doi: 10.3390/ijms27010157 (PMC12786108; doi:10.3390/ijms27010157)
Supplement: Supplementary file 1 [file ijms-27-00157-s001.zip › Supplement Figure.pdf]

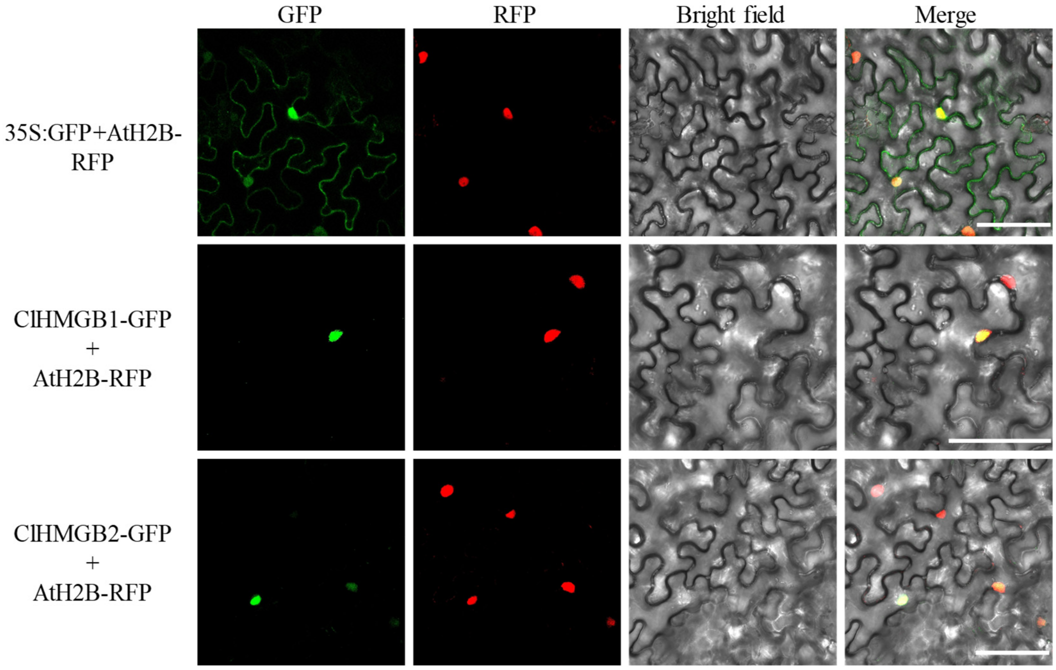

Figure S1. Subcellular localization of ClHMGB proteins in tobacco leaf epidermal cells. The 35S:GFP, ClHMGB1-GFP, and ClHMGB2-GFP constructs were individually co-expressed with the AtH2B-RFP through transient transformation. 35S:GFP and AtH2B-RFP served as negative and positive controls, respectively. GFP, green fluorescent protein; RFP, red fluorescent protein. Scale bar = 73 μm.

(a)

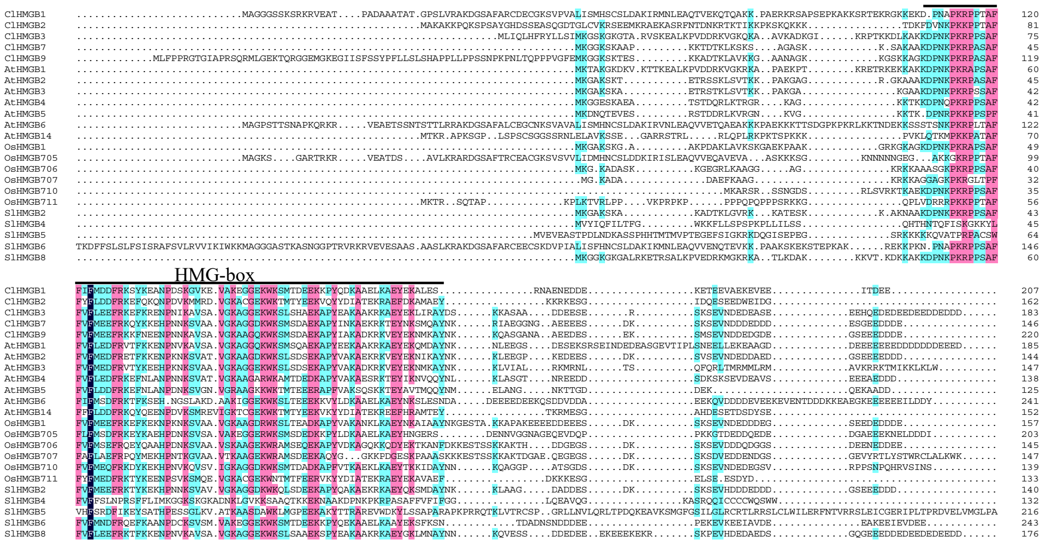

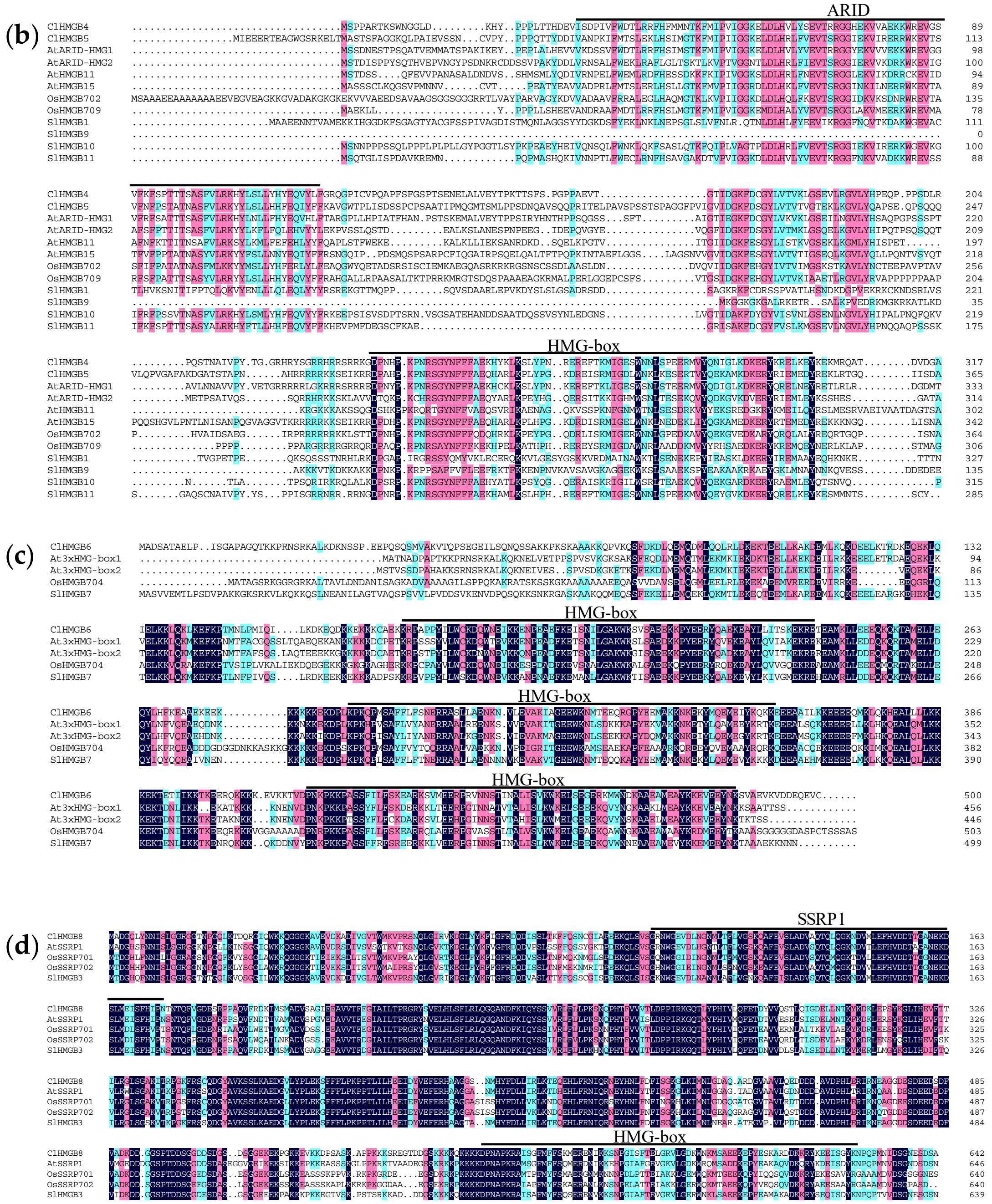

Figure S2. Multiple sequence alignment of HMGB proteins from four plant species. (a) HMGB class. (b) ARID-HMG class. (c) 3xHMG-box class. (d) SSRP1 class. Conserved domains (HMG-box, ARID, and SSRP1) are indicated by black lines. Species abbreviations: Cl, *Citrullus lanatus* (watermelon); At, *Arabidopsis thaliana*; Os, *Oryza sativa* (rice); Sl, *Solanum lycopersicum* (tomato).
